# Deep learning based discrimination of soft tissue profiles requiring orthognathic surgery by facial photographs

**DOI:** 10.1038/s41598-020-73287-7

**Published:** 2020-10-01

**Authors:** Seung Hyun Jeong, Jong Pil Yun, Han-Gyeol Yeom, Hun Jun Lim, Jun Lee, Bong Chul Kim

**Affiliations:** 1 Safety System Research Group, Korea Institute of Industrial Technology (KITECH), Gyeongsan, Korea; 2 Department of Oral and Maxillofacial Radiology, Daejeon Dental Hospital, Wonkwang University College of Dentistry, Daejeon, Korea; 3 Department of Oral and Maxillofacial Surgery, Daejeon Dental Hospital, Wonkwang University College of Dentistry, Daejeon, Korea

**Keywords:** Dentistry, Medical imaging, Mathematics and computing

## Abstract

Facial photographs of the subjects are often used in the diagnosis process of orthognathic surgery. The aim of this study was to determine whether convolutional neural networks (CNNs) can judge soft tissue profiles requiring orthognathic surgery using facial photographs alone. 822 subjects with dentofacial dysmorphosis and / or malocclusion were included. Facial photographs of front and right side were taken from all patients. Subjects who did not need orthognathic surgery were classified as Group I (411 subjects). Group II (411 subjects) was set up for cases requiring surgery. CNNs of VGG19 was used for machine learning. 366 of the total 410 data were correctly classified, yielding 89.3% accuracy. The values of accuracy, precision, recall, and F1 scores were 0.893, 0.912, 0.867, and 0.889, respectively. As a result of this study, it was found that CNNs can judge soft tissue profiles requiring orthognathic surgery relatively accurately with the photographs alone.

## Introduction

Dentofacial dysmorphosis (DFD) manifests itself in various aspects, including facial asymmetry, retrognathism, and prognathism^1^. Various orthognathic surgery techniques are used to solve these skeletal problems^2^. And diagnosis is important for DFD because it can predict the need for surgery^3^. Meanwhile facial photographs of the subjects are often used in the early stages of the diagnosis process. In medical and dental field, deep learning network could be used for screening or provisional diagnosis rather than final diagnosis and final treatment plan. If patients complaining DFD can be automatically evaluated whether orthognathic surgery is needed by plain facial photographs, the deep learning network can be used as a useful screening tool in dental field.

Convolutional neural networks (CNNs) continue to advance and are being applied in a variety of dental and maxillofacial fields. This can perform radiographic detection of periodontal bone loss^4^ and be used to diagnose cystic lesions using panoramic and cone beam computed tomographic images^5^. Survival prediction of oral cancer patients are also possible^6^. Perioperative blood loss in orthognathic surgery can be predicted^7^. Automated skeletal classification is possible by lateral cephalometry^8^.

However, to the best of our knowledge, CNNs have not been applied to the diagnosis of DFD using photographs of the subjects. Therefore, the aim of this study was to determine whether CNNs can judge soft tissue profiles requiring orthognathic surgery using facial photographs alone.

## Results

The classification results for the dataset are shown in the confusion matrix in Table 1. 366 of the total 410 data were correctly classified, yielding 89.3% accuracy. The values of accuracy, precision, recall, and F1 scores were 0.893, 0.912, 0.867, and 0.889, respectively. In addition, the visualization map of the classified pictures is overlapped with the original image. Lip, teeth, and chin are highlighted in the visualization map. Detailed photographs of subjects are not shown for anonymization.

**Table 1:** Classification results.

	Group I (ground truth)	Group II (ground truth)
Normal (prediction)	177 (true positive; TP)	17 (false positive; FP)
Surgery (prediction)	27 (false negative; FN)	189 (true negative; TN)

## Discussion

The CNNs used in this study has two main characteristics. First, the backbone network extracts feature maps independently from the front and side facial photographs. And inference can be performed synthetically by combining the feature vectors before entering the fully connected layer. Second, high-level feature maps created after passing through the backbone network are vectorized through global pooling. So even if the size of the image is different, inference is possible.

Global pooling layer is a method recently used to replace the classifier structure that combines the composite product layer and the fully connected layer^9^. The fully connected layer has a number of parameters, which require a long calculation time and a fixed input dimension. Therefore, this study used global pooling to eliminate this drawback.

The VisualBackProp method is used for visualization^10^. This is averaged in the depth direction from the extracted feature map after passing through each pooling layer. And it starts with the final pooling map and deconvolutions to the original size of the image, multiplying the average feature map to visualize it. Lip, teeth, and chin are highlighted in the visualization map. As with maxillofacial surgeons, CNNs also observed these sites and evaluated the need for orthognathic surgery.

In general, when diagnosing DFD through photographs, the facial part is considered and evaluated. In addition, when taking a photograph of the patient’s face, all patients did not take the same range, therefore, the study was conducted by cropping only the facial area. Detailed photographs of subjects are not shown for anonymization.

Meanwhile, analysis of this study was based only on facial esthetics, from facial photos in two planes, without any functional consideration such as occlusion, breathing, and temporomandibular disorder, etc. This was the setting for the sake of the methodology. However, this is a very limited vision at the time of deciding whether an orthognathic surgery is required or not for a specific patient. Indeed, vast majority of patients referring to orthognathic surgeon present some degree of malocclusion, even if their main demand is esthetic.

However, this study revealed that CNNs can intuitively determine the necessity of orthognathic surgery to some extent by looking at facial photographs. This is the difference between human and CNNs. On the other hand, the analysis of this study revealed a certain level of false positives and false negatives. This is also a limitation of the number of samples and the backbone network, but it can be interpreted as a result reflecting that soft tissue profiles alone cannot be used to determine all cases requiring orthognathic surgery.

Facial photographs of the subjects are commonly used in the diagnosis process of orthognathic surgery. As a result of this study, it was found that CNNs can judge soft tissue profiles requiring orthognathic surgery relatively accurately with the photographs alone.

## Materials and methods

### Datasets

In this study, transverse and longitudinal facial photographs of 822 patients who visited Daejeon Dental Hospital, Wonkwang University between January 2007 and December 2019 complaining DFD and/or malocclusion were used for training and testing a deep learning model. (mean age: 23.5 years, age range: 19 to 28 years, 452 males, 370 females) Adolescents with incomplete facial growth or those with congenital deformity, infection, trauma and tumor history were excluded.

Facial photographs of front and right side were taken from all patients. To classify patients about the need for surgery, posteroanterior and lateral cephalometry were obtained using Planmeca Promax (Planmeca OY, Helsinki, Finland). All radiographic images were evaluated by two orthodontists, three maxillofacial surgeons and one maxillofacial radiologist. Point A-nasion-point B (ANB) and Wits appraisal were used for diagnosing the sagittal skeletal relationship. Jarabak’s ratio and Björk’s sum were used for determining the vertical skeletal relationship.

With consensus of 6 specialists, patients who did not need orthognathic surgery were classified as Group I (411 subjects). Group II (411 subjects) was set up for the patients requiring orthognathic surgery due to skeletal problems such as facial asymmetry, retrognathism, and prognathism. Although many factors other than the skeletal part (patient’s preference, patient's soft tissue type, operator's preference, etc.) are considered to determine the final treatment plan, this study classified patients about the need for surgery only by the skeletal factors obtained by cephalometry. Other than the facial photographs, no other diagnostic data were used to classify the two groups by CNNs.

### Preprocessing and image augmentation

The photographs were cropped based on the forehead, ear, and chin of subjects and used for training and test. The purified datasets were divided into training and test sets using a random sampling method so that the ratio of group I and group II was close to 1:1 (Table 2). Image augmentation was performed to help generalize learning. Since geometric information about face contours is important, the data set is 9 times larger than the original by resizing at intervals of 0.2 to 0.9 times with the same horizontal and vertical ratios. This was doubled through left and right flips, creating a total of 18 times the dataset.

**Table 2:** Dataset configuration.

	Training	Test
Group I (normal)	207	204
Group II (surgery)	205	206
Total	412	410

### Architecture of the deep convolutional neural network

**Figure 1:**
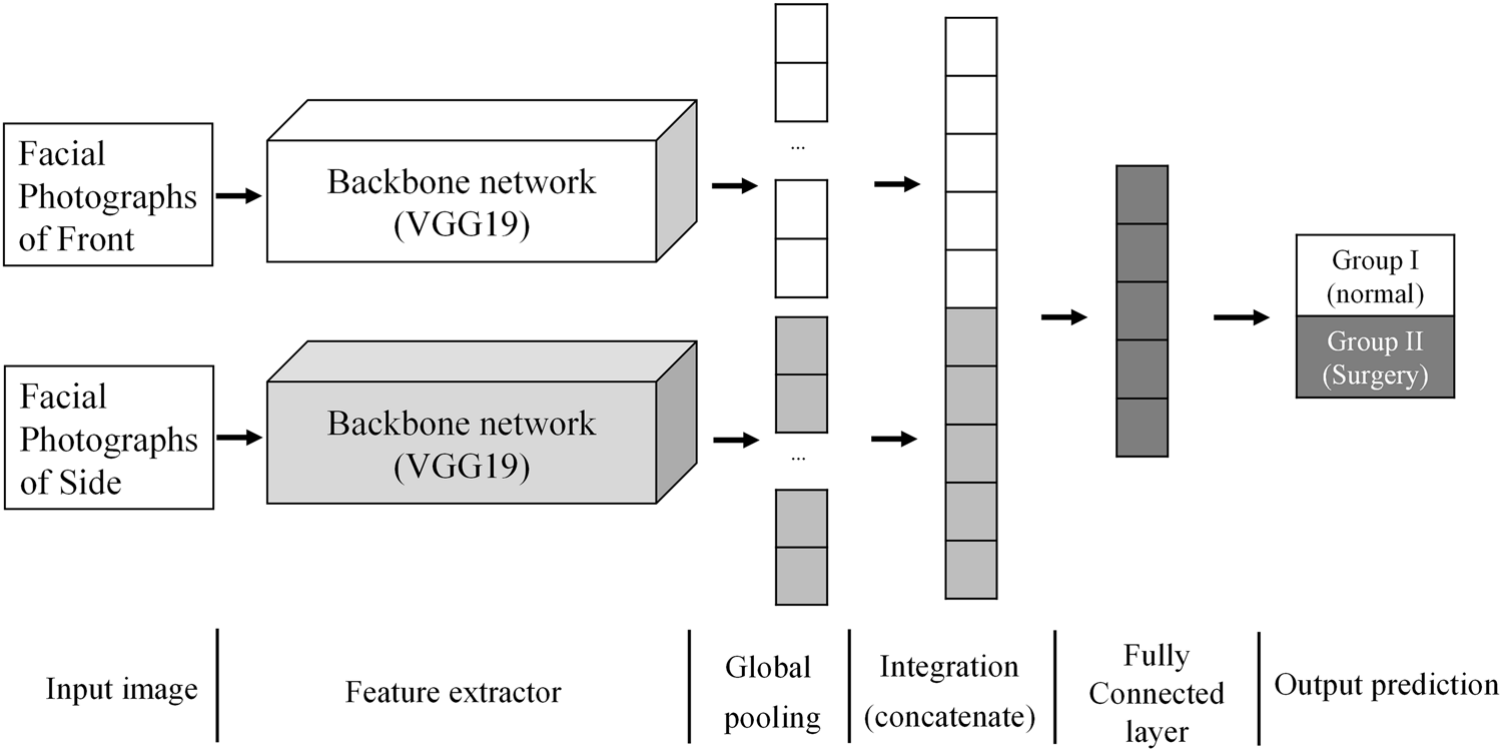
CNNs classifier using independent front and lateral facial photographs.

The CNNs used in this study is shown in Fig. 1. In order to determine the orthognathic surgery necessity, two independent facial photographs of the front and right side of the patient were used as input of CNNs. The proposed CNNs consists of the part of extracting the feature map from each of the front and side facial photographs, the part of vectorizing the extracted feature map, the part of combining the feature vectors, and the fully connected layer.

**Figure 2:**
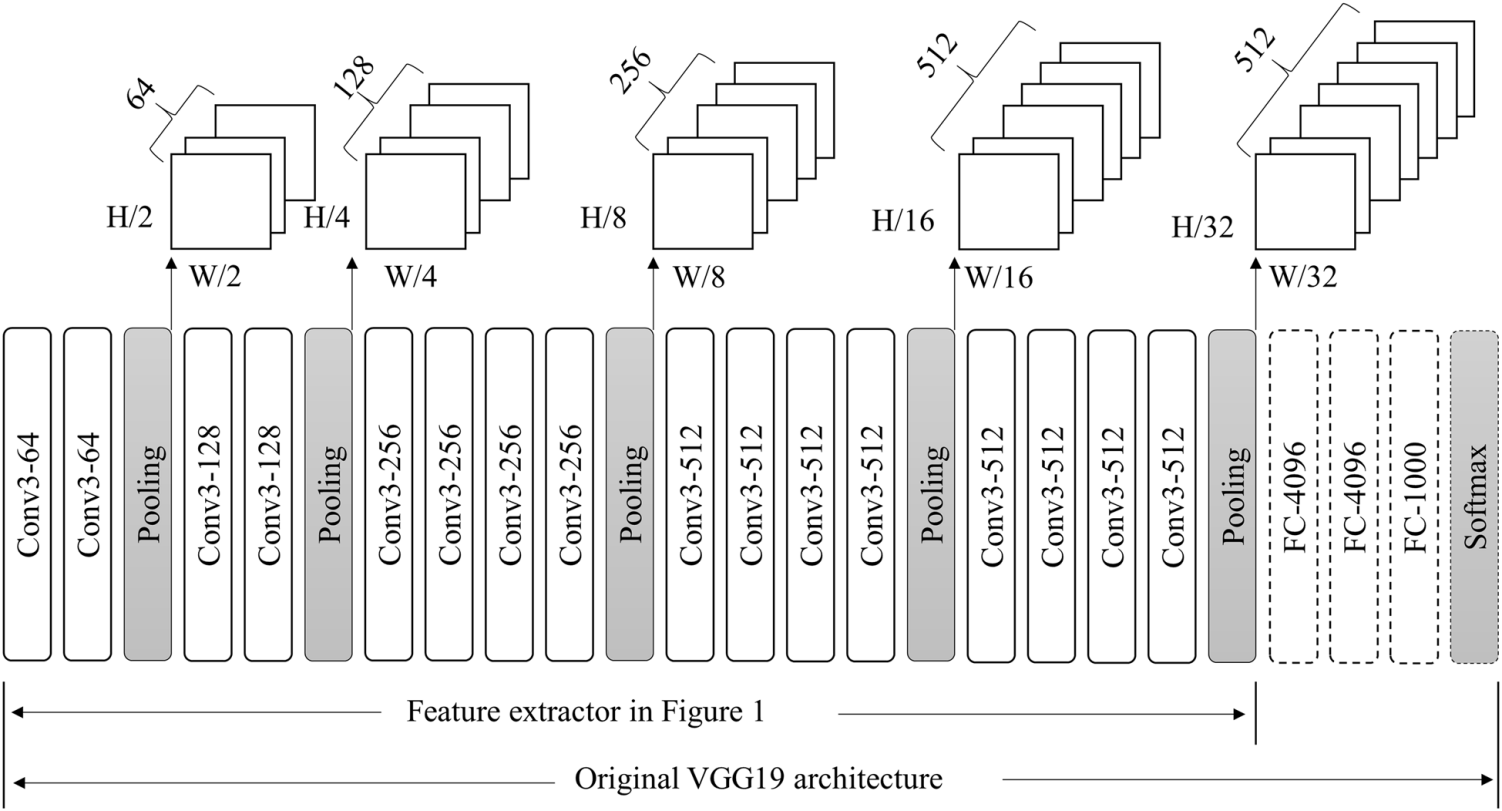
Feature extractor architecture using VGG19 network.

In this study, the CNNs of VGG19 was used^11^. This model increases the recognition rate by deepening the CNNs. It passes through 19 weight layers. In this study, 16 weight layers are used since fully connected layer is excluded (Fig. 2). It has 16 convolutional layers and 5 pooling layers. The width, height, and depth dimensions of the final feature map are determined by the shape of the composite product neural network and the pooling layer. We used 3 by 3 product and 2 by 2 max pooling. Therefore, passing through the feature map extraction layer reduces the width and height by 32 times and extracts the final feature map with a depth of 512.

**Figure 3:**
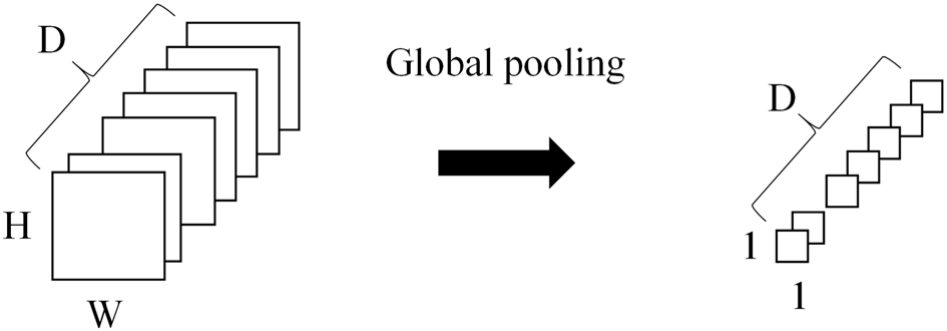
Global pooling.

The extracted feature map is converted into a feature vector through the global pooling layer. The average value of each feature map was calculated and vectorized in the depth direction of the feature map generated by the feature extractor (Fig. 3). The feature vectors generated for each image are combined as shown in Fig. 1 to classify groups through the fully connected layer. For the feature extractor, various models such as VGG^11^, ResNet^12^, DenseNet^13^, and so on can be used. The accuracies according to various backbone network were summarized in the supplement.

### Visualization method

**Figure 4:**
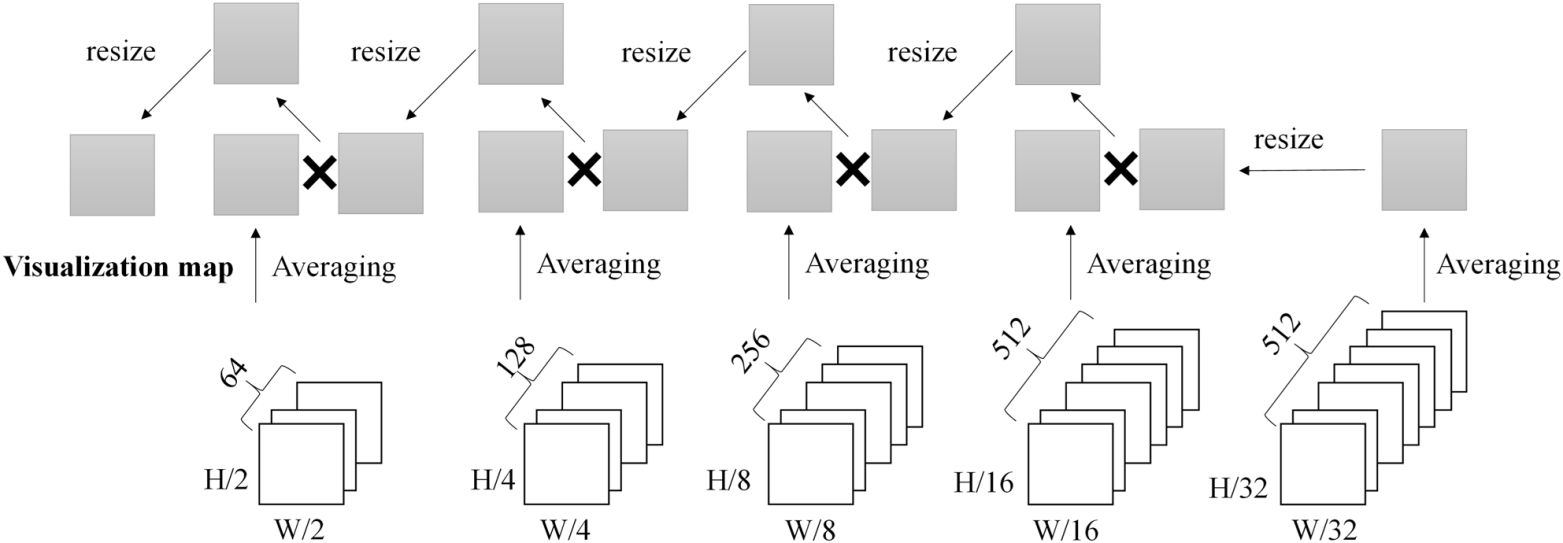
Feature visualization based on VisualBackProp.

The trained machine learning model was visualized so that the extracted part of the feature map of the front and side photographs could be identified. The VisualBackProp method was applied (Fig. 4)^10^. In this study, the final visualization map was created by resizing the pooling map instead of deconvolution.

Adam optimizer was used for learning^14^. The size of the batch size is 1 and the learning rate is 0.00001. The number of epochs is fixed at 10 and no learning decay is used. Network weights were initialized to the values of the pre-trained VGG19 model. And Tensorflow was used for training and testing.

### Statistical analysis

Statistical analysis was performed by calculating accuracy, precision, recall, and F1 score based on the confusion matrix shown in Table 1. The accuracy, precision, recall, and F1 scores were calculated by the following equations.

$$Accuracy=\frac{TP+TN}{TP+TN+FN+FP}$$

$$Precision= \frac{TP}{TP+FP}$$

$$Recall= \frac{TP}{TP+FN}$$

$${F}_{1, score} =2\times \frac{Precision\times Recall}{Precision+Recall}$$

### Ethical approval and informed consent

This study was performed within the guidelines of the World Medical Association Helsinki Declaration for biomedical research involving human subjects and was approved by the Institutional Review Board of Daejeon Dental Hospital, Wonkwang University (W2002/001-001). Informed consents were obtained from the subjects.

## Supplementary information

Supplementary Information.

## Data Availability

Data used in this study can be made available if needed within data protection regulation boundaries.
