# Supplementary material for: Deep learning based discrimination of soft tissue profiles requiring orthognathic surgery by facial photographs
Source: Sci Rep. 2020 Oct 1;10:16235. doi: 10.1038/s41598-020-73287-7 (PMC7529761; doi:10.1038/s41598-020-73287-7)
Supplement: Supplementary file 1 — Supplementary Information. [file 41598_2020_73287_MOESM1_ESM.docx]

Supplement

*Classification results according to gender*

The dataset was divided into male and female. The proposed classification method was applied to male and female datasets separately. The composition of dataset and corresponding accuracies are described in the table S1 and S2. When training is performed by dividing the dataset, the number of data available for training per model decreases, therefore reducing the overall accuracy. It showed better classification accuracy for the male with more data than female.

Table S1. Dataset configuration for female and male

|  |  | Training | Test |
| --- | --- | --- | --- |
| Female | Normal | 100 | 100 |
|  | Surgery | 100 | 98 |
| Male | Normal | 105 | 104 |
|  | Surgery | 107 | 108 |

Table S2. Classification accuracies for female and male.

|  | Accuracy (%) |
| --- | --- |
| Female | 78.7 |
| Male | 83.0 |

*Classification results according to various backbone network*

The proposed classification problem was solved by changing backbone network to VGG, ResNet, and DenseNet. Because the methodology applied for division of the dataset into the training and test set part leads to results bias and overoptimistic results, the dataset has been randomly divided into 412 and 410 for training and test respectively, 10 times and derive the accuracy accordingly. Note that, the numbers of Group I and II are also divided randomly. As shown in the Table S3 and Figure S1, over 84% accuracy was achieved on average across all backbone networks.

Table S3. Classification accuracy by various backbone networks.

|  | Accuracy | | |
| --- | --- | --- | --- |
| Model | mean | Best | Worst |
| VGG16 | 87.5 | 89.5 | 85.6 |
| VGG19 | 87.2 | 91.7 | 82.2 |
| ResNet18 | 88.5 | 93.2 | 86.8 |
| ResNet34 | 86.1 | 91.2 | 76.6 |
| ResNet50 | 84.4 | 89.5 | 77.1 |
| DenseNet121 | 88.4 | 90.0 | 83.9 |
| DenseNet161 | 87.6 | 91.2 | 83.9 |

Figure S1. Accuracies according to various backbone network with randomly divided dataset.
